# Indocyanine Green Fluorescence Angiography to Assess Tissue Perfusion Before Common Femoral Artery Aneurysm Ligation After Transfemoral Amputation

**DOI:** 10.1016/j.ejvsvf.2025.01.003

**Published:** 2025-01-27

**Authors:** Steven J.G. Leeuwerke, Harry G.M. Vaassen, Robbert Meerwaldt

**Affiliations:** a Department of Vascular Surgery, Medisch Spectrum Twente, Enschede, the Netherlands; b Multimodality Medical Imaging Group, Technical Medical (TechMed) Centre, University of Twente, Enschede, the Netherlands

**Keywords:** Common femoral artery, Indocyanine green fluorescence angiography, Peripheral aneurysm

## Abstract

**Introduction:**

The superiority of indocyanine green fluorescence angiography (ICG-FA) to the clinical eye alone to assess tissue perfusion has been demonstrated in various surgical fields. This short report demonstrates the *in vivo* use of ICG-FA to assess skin perfusion before ligating the external iliac artery (EIA) to exclude a common femoral artery (CFA) aneurysm.

**Report:**

A 70-year-old man presented with a CFA aneurysm after a previous transfemoral amputation. Ligation of the EIA was proposed, but concerns about tissue perfusion warranted a careful approach. The CFA was exposed using an infra-inguinal incision. Intra-operative ICG inflow and washout patterns were semi-quantitatively analysed to assess dermal perfusion of the femoral stump before and after EIA clamping. Based on similar patterns, distal EIA ligation was performed without ischaemic complications.

**Conclusion:**

Indocyanine green fluorescence angiography is a promising technique for *in vivo* assessment of tissue perfusion in peripheral arterial disease, but standardised protocols for perfusion quantification are required to more accurately predict tissue viability.

## Highlights

• Indocyanine green fluorescence angiography (ICG-FA) allows semi-quantitative assessment of tissue perfusion.
• ICG-FA has been proven to be superior to the clinical eye alone in various surgical fields.
• The subjective interpretation of images and lack of benchmark values remain challenging in the clinical applicability of ICG-FA.

## Introduction

Since the development of indocyanine green (ICG) for photography during World War II, its near-infrared (NIR) fluorescent properties and the availability of high-definition NIR cameras have recently led to the widespread application of ICG fluorescence angiography (ICG-FA). The superiority of ICG-based perfusion assessment to the clinical eye alone has been demonstrated in several surgical fields, including the visualisation of biliary anatomy during cholecystectomy and anastomosis perfusion assessment in colorectal surgery.^1^, ^2^, ^3^, ^4^ Because ICG can be used liberally due to its low cost and non-toxic qualities,^2^ new promising applications are emerging, such as assessing bowel viability after revascularisation in mesenteric ischaemia and predicting the success of peripheral revascularisation.^2^, ^3^, ^4^, ^5^, ^6^, ^7^, ^8^

This short report describes the case of a 70-year-old man with an asymptomatic common femoral artery (CFA) aneurysm after a previous transfemoral amputation (TFA), in which ICG-FA was used to successfully predict tissue viability before external iliac artery (EIA) ligation.

## Report

A 70-year-old diabetic and hypertensive man presented to the Emergency Department with a slowly expanding and pulsatile, but otherwise asymptomatic mass in the left groin. His vascular history consisted of a ruptured juxtarenal abdominal aortic aneurysm in 2015, for which he underwent an emergency aortic replacement using a bifurcated graft placed through an open approach. The post-operative course was complicated by ischaemic colitis of the descending and sigmoid colon, for which a left hemicolectomy and colostomy were performed. In addition, he developed ischaemia of the left lower extremity due to dissection with stenosis at the level of the iliac anastomosis, with subsequent occlusion of the left graft limb. Despite successful endovascular embolectomy with relining of the graft limb, progressive ischaemia and necrotising fasciitis necessitated a TFA. He eventually recovered.

**Figure 1:**
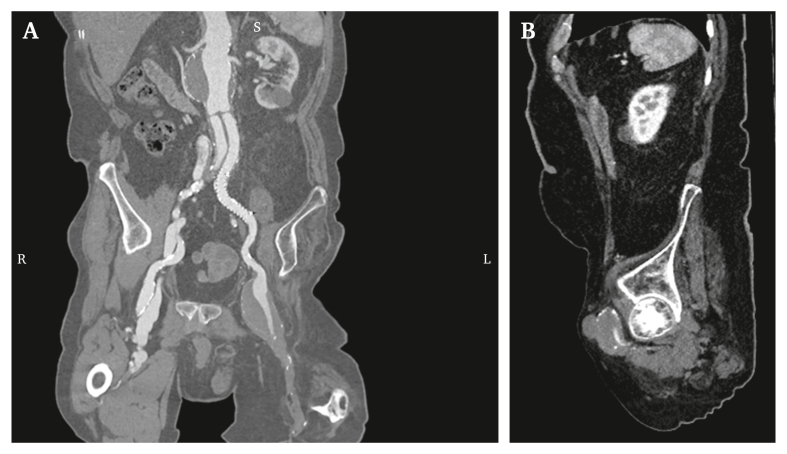
Coronal multiplanar reconstruction of the pre-operative computed tomography angiogram, illustrating a left common femoral artery aneurysm with an occluded superficial femoral artery (A) and a proximally patent but small profunda femoris artery (2–3 mm) on sagittal view (B).

Years later, the patient returned to the Emergency Department with a slowly expanding and pulsatile, but otherwise asymptomatic, mass in the left groin. Computed tomography angiography (CTA) revealed a CFA aneurysm with a maximum diameter of 53 mm and a saccular component on the anterior side (Fig. 1). The CFA aneurysm extended up to the EIA without signs of impending rupture, and the superficial femoral artery was occluded. The profunda femoris artery (PFA) originated from within the aneurysm and appeared patent proximally, with a diameter of 2–3 mm (Fig. 1b). Distal patency was difficult to assess; therefore, it was suspected that the PFA had a minimal contribution to stump perfusion. The CTA showed no complications besides progression of the juxtarenal aneurysm to 66 mm proximal of the aortic bifurcation graft.

After a multidisciplinary team meeting, various options were considered. Given his age and comorbidities, EIA ligation through a small infra-inguinal approach was deemed to be the simplest solution with the least chance of complications and future interventions. A supra-inguinal extraperitoneal approach to avoid the groin was considered, but an infra-inguinal approach was deemed the most straightforward strategy based on pre-operative images. Nonetheless, the impact of EIA ligation on stump perfusion was uncertain. Intra-operative ICG-FA was proposed to assess dermal stump perfusion before and after EIA clamping. The team was prepared for a same session ilioprofunda graft interposition if there was a significant perfusion deficit. Possible alternative strategies included an ilioprofunda endovascular bypass through retrograde access from the aneurysm, or antegrade through access from the right CFA.^9^ Besides being technically more challenging, the absence of arterial outflow due to the TFA and questionable distal PFA patency posed a high risk of occlusion of the endovascular bypass and subsequent ischaemia compared with simple suture ligation of the EIA. Another possible strategy was coil embolisation of the EIA using Amplatz plugs through retrograde access from the aneurysm after balloon occlusion to facilitate ICG perfusion measurements. However, this method would have subjected the patient to unnecessary radiation exposure and incurred higher costs.

**Figure 2:**
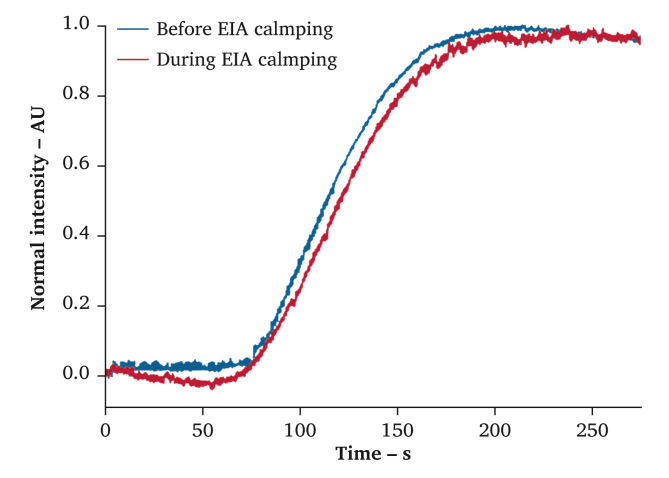
Intensity-over-time curves extracted from near-infrared fluorescence measurements before and after external iliac artery ligation. Intensity values are normalised with respect to the maximal value. Injection of ICG occurred at t = 0.

**Figure 3:**
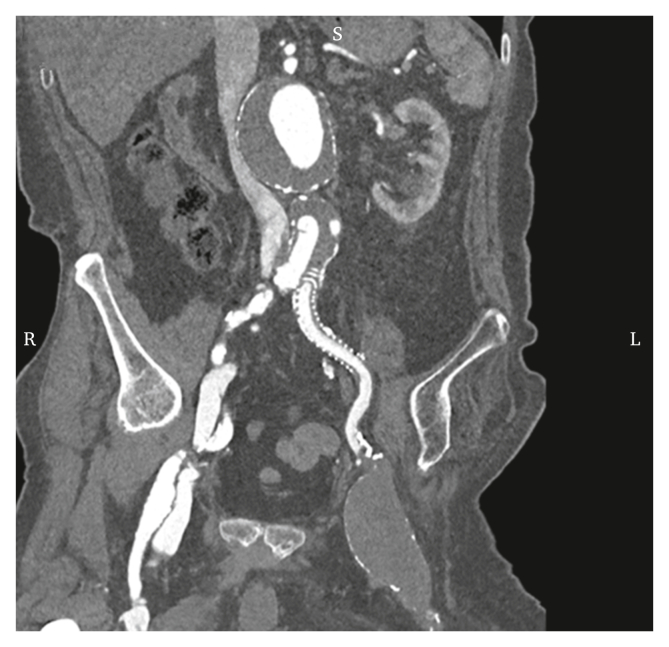
Coronal multiplanar reconstruction of the post-operative computed tomography angiogram, demonstrating successful ligation of the external iliac artery and exclusion of the common femoral artery aneurysm.

Under general anaesthesia, the EIA was exposed just proximal to the CFA aneurysm using an infra-inguinal approach with manual traction by an assistant without the need for a retractor. The ICG-FA was performed using the Rubina imaging system (IMAGE 1 S 4U Rubina, Karl Storz SE & Co. KG, Tuttlingen, Germany) in monochromatic NIR mode, placed approximately 15 cm from the skin. Then, 5 mg of ICG (Infracyanine, Fresenius Kabi AG, Bad Homburg, Germany) was administered intravenously and flushed with 10 mL of saline. The dermal ICG inflow and washout patterns were observed for five minutes and semi-quantitatively examined using in-house developed software (Fig. 2). A more detailed description of this method is presented in the supplementary material of Vaassen *et al.* 2022.^10^ Before clamping, the first rise in fluorescence was observed 65 seconds after injection, while peak fluorescence intensity was reached after 200 seconds. The measurement after EIA clamping displayed the first rise after 67 seconds and peak fluorescence after 205 seconds. Based on these findings, it was concluded that the PFA barely contributed to stump perfusion and that the distal EIA could be safely ligated. The patient was discharged the next day. The post-operative course was complicated by a wound infection, which was resolved with antibiotics, but remained otherwise uneventful up to eight months post-operatively. During follow-up, the patient experienced no pain or signs of stump ischaemia. Post-operative CTA showed successful exclusion of the CFA aneurysm (Fig. 3).

## Discussion

This case demonstrates how ICG-FA can predict the physiological effect of ligating a major vessel on tissue viability to treat a CFA aneurysm. It is believed that this application of the technique has not been described previously. Fluorescence-guided surgery is increasingly being used for various indications at this centre. Reversible bowel ischaemia can be difficult to visually assess in patients with mesenteric ischaemia, and ICG-FA can help prevent unnecessary resections while ensuring complete removal of devitalised tissue.^7^ Similarly, the use of ICG-FA during colorectal surgery has been shown to reduce the rate of anastomotic leakage.^3^^,^^4^ In selected cases, ICG-FA can also be used to enhance visualisation of the biliary tract during laparoscopic cholecystectomy and of lymph nodes during sentinel node procedures. Further studies into its value in peripheral arterial occlusive disease are currently being conducted. Most modern laparoscopic imaging systems incorporate a monochromatic NIR mode and a flask of 25 mg infracyanine green is priced around €85. Therefore, due to its low costs and non-toxic characteristics, ICG-FA is perceived as an accessible and versatile intra-operative tool. It can be relied upon in routine procedures such as sentinel node biopsy but can also provide additional information in unusual cases like the one presented here.

A challenge and limitation of ICG-FA perfusion assessment is the subjective interpretation of images and the lack of benchmark values. Previous studies using ICG-FA in lower extremity arterial disease show that fluorescence patterns are immediately affected by revascularisation.^6^^,^^8^ Therefore, in this case, the safety of EIA ligation could be accurately predicted by comparing dermal stump perfusion before and after clamping. Moreover, the authors chose to evaluate the data based on time-based dynamics, to avoid factors like camera distance and ambient light, which influence the intensity values and are hard to reproduce. To make ICG-FA even more powerful in the real-time assessment of tissue perfusion, it is imperative that larger clinical studies provide objective benchmark values for patients with perfusion deficits.

### Conclusion

Indocyanine green fluorescence angiography is a promising technique for the *in vivo* assessment of tissue perfusion in peripheral arterial disease. However, standardised protocols for perfusion quantification are required to more accurately predict tissue viability.

## Funding

This research did not receive any specific grant from funding agencies in the public, commercial, or not-for-profit sectors.

## Conflict of interest

None.
